# Are matrix metalloproteinases relevant therapeutic targets for prostate cancer bone metastasis?

**DOI:** 10.3747/co.v15i4.216

**Published:** 2008-08

**Authors:** R.D. Bonfil, R. Fridman, S. Mobashery, M.L. Cher

**Affiliations:** * Department of Urology, Wayne State University School of Medicine, and The Barbara Ann Karmanos Cancer Institute, Detroit, MI, U.S.A.; † Department of Pathology, Wayne State University School of Medicine, and The Barbara Ann Karmanos Cancer Institute, Detroit, MI, U.S.A.; ‡ Department of Chemistry and Biochemistry, and the Walther Cancer Research Center, University of Notre Dame, Notre Dame, IN, U.S.A.

**Keywords:** Matrix metalloproteinases, prostate cancer, bone metastasis

## INTRODUCTION

After basal and squamous cell skin cancers, prostate cancer is the most frequent cancer in men in the United States, with 186,320 men estimated to be diagnosed with the disease and 28,660 expected to die from it in 2008 1. Approximately 90% of hematogenous metastases in prostate cancer patients occur in bone 2, making skeletal complications the leading cause of morbidity and mortality in these patients.

Although skeletal metastases from prostate cancer are usually considered osteoblastic by radiologic analysis, histologic and biochemical studies suggest that osteolytic and osteoblastic responses are sequentially linked 3–5. A vicious cycle in which prostate cancer cells secrete factors that stimulate bone matrix turnover, which in turn releases growth factors that enhance tumour proliferation, has been proposed 6. In the normal bone microenvironment, endosteal cells (osteoblasts, osteoclasts) and bone marrow cells (fibroblasts, pre-osteoblasts, pre-osteoclasts, lymphoid and myeloid cells, endothelial cells, hematopoietic and mesenchymal stem cells) communicate with each other in a homeostatic process that involves transmission of various biologic signals. However, this delicate equilibrium is perturbed when prostate cancer cells colonize the bone, leading to an alteration of the physiologic balance between bone formation and bone resorption, and to a hospitable environment for expansion of the metastases 7.

A role for matrix metalloproteinases (mmps) in bone metastasis has long been suspected. Because mmps contribute to the process of normal bone remodelling, and because enhanced turnover of bone matrix occurs when tumour cells metastasize to bone, a role can be predicted for these enzymes in metastasis-associated bone modification.

## MMPs AT A GLANCE

The mmp family of Zn^2+^-dependent endopeptidases comprises at least 24 members, which have the combined capacity to degrade virtually all the structural components of extracellular matrix [ecm (for review see 8,9)]. Most mmps are secreted, although 6 of them are membrane-tethered (mt) proteins that have the ability to mediate proteolytic events at both the cell surface and in the immediate pericellular milieu. The mmps consist of an N-terminal signal sequence, a propeptide domain, a catalytic domain, and a C-terminal domain known as the hemopexin-like domain. In the case of the mt-mmps, a C-terminal transmembrane domain or a glycosylphosphatidylinisotol link domain is added.

All mmps are synthesized as inactive zymogens and are maintained as such by coordination of a cysteine residue within the propeptide domain, with a Zn^2+^ ion present in the catalytic domain, which prevents binding and cleavage of the substrate. The dissociation of cysteine from the Zn^2+^ atom, known as “cysteine switch,” is a complex process, the details of which have emerged only recently^10^ The activity of mmps is specifically inhibited by tissue inhibitors of mmps (timps: timp-1, timp-2, timp-3, and timp-4), which usually bind to the active species, inhibiting catalysis. In some mmps, the timps can bind to the zymogens.

For a long time, the significance of the binding of certain timps to the latent form of some mmps was unclear. It is now known that, for example, low concentrations of timp-2 can promote activation of pro-mmp-2 through a mechanism in which timp-2 acts as a linker between the pro-mmp-2 zymogen and mt1-mmp at the cell surface. In this way, the propeptide of the pro-mmp-2 present in this ternary complex formed on the cell surface is cleaved by an adjacent active mt1-mmp. Conversely, high levels of timp-2 relative to mt1-mmp would inhibit activation by blocking all free mt1-mmp molecules.

Initially, mmps were recognized as proteinases exclusively committed to ecm remodelling during invasive, angiogenic, and metastatic processes. They are now known to have additional roles, such as activation of growth factors, cleavage of cell surface receptors, release of angiogenic factors, release of apoptotic ligands, and generation of angiostatic molecules, to mention just a few. These multiple roles demonstrate the complexity and multiplicity of the substrates of mmps and their ample range of biologic activity in normal and pathologic conditions alike.

## MMPs AND PROSTATE CANCER BONE METASTASES

Cancer cells that disseminate to bone alter the normal skeletal remodelling process, upsetting the balance between bone formation and bone resorption. Because mmps are involved in the physiologic turnover of ecm and in bone remodelling, a contribution of mmps to prostate cancer bone metastasis seems to be reasonable. In fact, the participation of mmps in the pathogenesis of osteolytic prostate cancer bone metastasis was confirmed by several experimental studies.

More than 10 years ago, it was shown that the combined administration of alendronate (a bisphosphonate compound) and of paclitaxel inhibits bone metastases produced in severe combined immunodeficient (scid) mice by intravenous inoculation of human PC-3 ML cancer cells. This effect was mainly the result of a complete abrogation of the production and secretion of mmps 11. *In vitro,* it was found that PC-3 cells produce s when placed on bone surfaces 12, and secrete mmp and that *MMP2* and *MMP9* are among a set of genes altered when prostate cancer cells and bone marrow stromal cells interact 13. Those findings were further supported by studies in prostate cancer patients with skeletal metastases who had elevated serum levels of and mmp-2 mmp-9.^14^,^15^

Using the scid-hu model for bone metastasis, in which human prostate cancer cells are grown in human bone xenografts in scid mice 16, we confirmed high expression of mmp-2 and mmp-9 in cancer cells and in neighbouring bone stromal cells. Those data were consistent with our observations in bone biopsy specimens obtained from prostate cancer patients 17. Moreover, systemic administration to the mice of the broad-spectrum mmp inhibitor BB-94 (batimastat) inhibited the proliferation of prostate tumour cells growing within the human bone implants. That inhibition was accompanied by reduced degradation of bone marrow trabeculae and decreased osteoclast recruitment 17.

Recently, we showed an upregulation of net mmp-9 activity shortly after establishment of PC-3 cells in human bone xenografts—an increase that coincided with a wave of osteoclast recruitment and vigorous bone degradation 18. Experimentally, the activation of mmp-9 that occurs during prostate cancer–bone interaction is species-specific, because active mmp-9 was found when human prostate cancer cells grew within human bone tissue, but not when they grew within mouse bones 19.

Tumour-associated osteoclasts are known initially to dissolve the mineralized bone matrix by acid secretion and then to disrupt the exposed non-mineralized ecm by using proteases. Although mmp-9 does not degrade type i collagen, the most abundant organic component in bone, we found that the protease is expressed mainly by osteoclasts. That finding suggests that active mmp-9 induced by prostate cancer–bone interaction most likely contributes to osteolysis by indirect mechanisms. For instance, mmp-9 may cause the release of ecm-bound vascular endothelial growth factor into a soluble and bioactive form 20, thereby favouring the angiogenesis of intraosseous prostate tumours and the subsequent growth of those tumours. That effect ultimately stimulates new osteoclastic activity necessary to gain more space for expansion of the tumours.

The foregoing hypothesis was confirmed by experimental therapy with SB-3CT, a novel mechanism-based mmp inhibitor with high selectivity for mmp-9 21,22. This inhibitor is primed, in a chemical step, for potent inhibition of gelatinases once bound to the active sites of these enzymes. Treatment with SB-3CT of scid mice bearing prostate cancer bone tumours resulted in significant inhibition of angiogenesis and intraosseous tumour growth, together with reduction in osteolysis 23. Those results indicate an important contribution of mmp-9 to neovascularization of expanding bone metastases and to subsequent bone degradation. Moreover, pro-mmp-9 has been shown to play a crucial role in osteoclast recruitment 24. The existence of abnormalities in developmental angiogenesis and ossification in mice with null mutation in *MMP9* 24,25 further supports the importance of mmp-9 not only in normal but in pathologic processes occurring in the skeleton.

Membrane-tethered 1 mmp knockout mice also present severe skeletal abnormalities, mostly as a result of their incapacity to degrade crosslinked fibrillar type i collagen prevalent in the bone matrix^26^. In prostate cancer, mt1-mmp expression correlates with a more aggressive behaviour and has been shown to promote invasion and metastasis 27–29. Immunohistochemical studies of primary prostate adenocarcinomas revealed a heterogeneous pattern, with some malignant glands positive and others negative for mt1- mmp 30. That finding, together with a uniform and strong mt1-mmp expression observed in all cases of prostate cancer bone metastases analyzed 31, suggests the existence of a selective process in which mt1-mmp–expressing prostate cancer cells may have more tendency to metastasize to skeleton. Alternatively, the bone microenvironment might induce the expression of mt1-mmp in prostate cancer cells after their arrival in the marrow.

We conducted a series of studies to assess the contribution of prostate cancer cell–derived mt1-mmp to bone metastasis. We overexpressed mt1-mmp in LNCaP human prostate cancer cells (which have baseline non-detectable expression of the protease), while its expression was attenuated in DU145 cells (which have baseline high mt1-mmp expression) using small interfering rna. We showed that intratibial injection of those cells resulted in completely opposite phenotypes in terms of intraosseous tumour growth and bone response. Compared with controls, tibiae injected with LNCaP cells overexpressing mt1-mmp showed increased osteolysis (as demonstrated by radiography and histomorphometry) and enhanced intraosseous tumour growth. In contrast, mt1-mmp downregulation in high-expressing mt1-mmp DU145 prostate cancer cells led to diminished intraosseous tumour growth and a mixed bone reaction, in which osteosclerotic responses predominated 31. We further showed that mt1-mmp upregulation in cancer cells resulted in the release of one or more factors that promoted osteoclast differentiation *in vitro.* That effect was abrogated by pharmacologic inhibition of mt1-mmp activity and by osteoprotegerin, a soluble decoy receptor of the osteoclastogenic receptor activator of nuclear factor κB ligand (rankl). Our results strongly suggest the possibility that prostate cancer–associated mt1-mmp promotes an osteolytic response by shedding membrane-bound rankl (mrankl) from the cancer cell surface. Recently, mmp-7, produced mainly by osteoclasts at the prostate tumour–bone interface, has also been reported as a rankl “sheddase,” promoting osteolysis 32. Together, these data suggest that specific inhibition of certain mmps in prostate cancer bone metastasis may be therapeutically beneficial.

## NEW THERAPEUTIC CHALLENGES

Much of the initial excitement associated with the use of broad-spectrum mmp inhibitors in animal tumour models has been mitigated by a lack of therapeutic efficacy and undesired side effects observed in clinical trials with cancer patients 33–35. Some of the potential reasons for the failure of these agents include

- testing of patients with advanced high-volume disease refractory to other treatments;
- use of broad-spectrum inhibitors of mmps with unspecific and reversible effects;
- unknown repertoire of proteases expressed by the patients’ tumours (protease “degradome” 36) before and during treatment;
- unintended inhibition of mmps with important physiologic roles (anti-targets), probably resulting in neutralization of the effects of the inhibitors on actual mmp “targets” that truly contribute to disease 37;
- lack of studies to monitor mmp inhibition during treatment; and
- unknown effective doses of mmp inhibitors and diminished therapeutic index because of forced dose reduction to tolerable levels.

The first drugs developed for mmp inhibition were peptidomimetic hydroxamate compounds with potent inhibitory effects, but no selectivity (for example, batimastat). The second-generation mmp inhibitors exhibited some marginal selectivity (for example, prinomastat). Because those compounds failed in clinical trials, a third generation of selective mmp inhibitors is now being developed and considered for cancer therapy 38,39, aiming to obtain the maximal effect on the disease in which the mmp target is involved with minimal adverse reactions. These selective inhibitors are hoped to have a ratio of at least 1000 between the *K_i_* (the dissociation constant for binding of inhibitor) values for mmp anti-targets and those for mmp targets 38,39. These prospects should be explored and might prove efficacious, but even selectivity by a factor of 1000 might not solve the clinical riddle of compounds that serve as linear competitive inhibitors.

For example, potent linear competitive inhibition of target mmps at low nanomolar or picomolar levels *in vitro,* despite the “factor of 1000” selectivity, might not prove selective *in vivo* because a low micromolar or high nanomolar level of activity against the anti-target mmps will still foster *in vivo* consequences. The challenge is not necessarily an issue of affinity for the target, but rather of the mechanism for discrimination other than mere recognition events between the drug and the target. In that vein, the mechanism-based gelatinase inhibitor SB-3CT and its new structural variants stand out. This inhibitor class takes advantage of the active site of the enzyme to undergo a specific chemical transformation facilitated by the target enzyme itself. Whether a given mmp might be able to perform this reaction, or whether it might not, a process that leads to potent enzyme inhibition is at the root of its ability to serve as a selective mmp inhibitor to the given target. The concepts pertinent to inhibition of mmp have been discussed in a recent review 40.

In the particular case of prostate cancer patients, no clinical trials have been performed to evaluate the therapeutic efficacy of mmp inhibitors on bone metastasis. We believe that the knowledge obtained in recent years using animal models has provided validated mmp targets that, together with the development of third-generation mmp inhibitors, would justify use of those inhibitors in the treatment of prostate cancer patients with skeletal metastasis. For instance, prostate cancer patients with locally advanced disease who have a high probability of developing bone metastasis and no current prospect of curative treatment could benefit from therapy with novel mmp inhibitors.

For successful treatment, it is crucial that the protease degradome for tumours in each patient be assessed and that inhibition of the mmp being targeted be confirmed by appropriate methods during treatment. Alternatively, combination therapy using inhibitors for mmp targets and agents that can block osteoclastic action (such as bisphosphonates or anti-rankl drugs) could reasonably be employed in clinical trials involving prostate cancer patients with potential to develop bone metastases. The experimental evidence described herein showing a key role for mmps in bone metastasis suggests that targeting those mmps could have therapeutic value. New approaches must be explored, especially given that current approaches for treating bone metastasis in prostate cancer patients are still limited and only palliative.
